# The Seven Ages of Man

**Published:** 1888-10-06

**Authors:** 


### The Doctor's Pulpit.

### THE SEVEN AGES OF MAN.

### I.—The Infant.

"First the infant,
Mewling and puking in his nurse's arms."

Shakespeare does not seem to have been passionately fond of children. If he had been, he would not probably have immortalized them at such an unpleasant moment. Infancy is a well-marked and definite stage of human existence; but a well-conditioned mortal would not have thought that the most characteristic circumstance of infant life was one of "mewling and puking." "Mewling" everybody knows to be "whining," and the north countryman, at any rate, knows that "puking" means vomiting. We do not often find it necessary to quarrel with Shakespeare; but we must here affirm, and stoutly, that he has put a distinct libel upon infants. As to the "mewling," or "whining," a healthy and well- cared-for infant should whine very little, if at all. The natural tendency of the little creature is in a direction the very opposite of whining. No doubt if the nurse be one of those stupid antiquities who believe in tight body bandages, butter and sugar, and all the foolish abominations of the "bad old times," there may be much infantile discomfort and a good deal of consequent "mewling." Probably in Shakespeare's time nurses were of a lower and more ignorant type than even the famous "Sarah". and her dearly beloved "Betsy." If they were, we may perhaps believe the dramatist's experience of babies led him to conclude that the discription of them which would most readily commend itself to the frequenters of his playhouse was the very one he gave. The usual condition of the infant, according to him, was one of "mewling and puking in his nurse's arms."

To come down from the discursive and general to the definite and practical: This proposition is pretty constantly true—that a "whining" infant is an unhealthy infant. There is something wrong either with its constitution, or its food, or its clothing, or its general management. If the trouble be constitutional, measures should be taken, under the best medical guidance, to modify the constitutional state for the better. It is quite within the competency of medical treatment to improve the natural constitution. Probably no period of life is more amenable to judicious management than the infantile. The cause of the "mewling" may, however, be pretty generally looked for in the food. The feeding of an infant is a perfectly simple business if the mother happens to be healthy and sensible. But many mothers, particularly in towns, without being positively unhealthy, are feeble and debilitated. They cannot supply their infants with nourishment which is both good in quality and abundant in quantity. In such circumstances the little one cannot possibly be robust. Nothing is more certain than that the quality of a house will depend on the quality of the stone and lime and wood and other things out of which it is built. It is quite impossible to build a good house with poor materials. It is exactly so with the baby. The little creature can only work with the materials supplied to it. If they are poor and deficient, it will be poor and deficient too. Nothing can appear in the body which has not previously been in the nourishment it receives. Many people think that baby is bound to be all right so long as it is supplied with mother's milk. That, however, as we have shown, entirely depends upon the mother. If she be a feeble and sickly woman she can hardly do a worse thing for her baby than to nurse it herself.

The artificial feeding of babies is a duty requiring the utmost care and good sense. It is by no means a mysterious proceeding. Any woman of average ability can do it if she will. But the misfortune is that women of the baby-nursing class are so unreasoning and so obstinate. They often have one method, and one only. If it happen to suit a particular infant, well and good; that baby will thrive. But if a baby is met with which cannot prosper on the routine method, woe be to it. Then the "mewling and puking" begin. "Change the method," suggests an observer. "Never!" A downright pig-headed nurse—and there are many such—would far rather an infant died than she would disbelieve her own rules or try any one else's. A really reasonable woman is worth her weight in gold. Such a woman in the nursery is above all price. If "Robb's biscuits" do not agree with her charge, she will try "tops and bottoms." If "tops and bottoms" are heavy, "rusks" may be found suitable. If rusks fail, she will try Savory and Moore's, or some other prepared food. If the nursery milk is unsatisfactory, she will change it. If still it does not produce the desired result, she will change again, or try a "Swiss preparation." A determined woman of this kind will never be at rest until she has got some kind of food which both pleases the infant palate, agrees with its stomach, and makes it grow and thrive.

The clothing of babies must similarly be subject to the methods of commonsense. The great rule here is to consider the baby's comfort and safety. It is as shameful as it is silly to make a baby a milliner's model. Millinery, as it is now understood, should be banished from the nursery. If the writer could be a baby again, and carry his present experience with him, one of the first things he would do would be to organize a huge conspiracy of babies to burn down all the milliners' shops. Is it possible for any man or woman, inside or outside a lunatic asylum, to conceive anything more entirely destitute of a single grain of intelligence than the clothing of some babies? It is stiff where it should be yielding, tight where it should be loose, short where it it should be long, and long where it should be abbreviated. On certain parts of the body where clothing might be dispensed with, as the hands and feet, there are gloves and shoes and stockings such as make movement impossible. In certain other parts which should be warmly clothed, as the chest and shoulders, nudity is the fashion which women most delight in. But why repeat for the ten thousandth time these stale platitudes? For this reason, that women who will not obey the plain dictates of reason and experience may at least know that they are wicked fools.

"Puking" is undoubtedly one of the failings of many infants. It is not a very alarming occurrence if it be not too frequently repeated. The baby stomach is a highly sensitive organ, and often has the happy propensity of rejecting straightway all the food which is in excess of Nature's requirements. So long as the "puking" is of this kind, the mere throwing out of the excess, it is quite as it should be. The baby vomits, but thrives and grows. He should not then be dosed with physic or have his food changed. "Let well alone." But "puking" may be caused by acidity, or flatulence, or some other form of indigestion. It should then be attended to and cured. Sometimes a change of food is all that is needed. When this fails, medical aid should be at once sought. Parents ought to remember that not only are they building up their child's constitution, but also that their child is building up his own. Now, the digestive organs are the child's building hands, his trowel, his mallet, and indeed all his tools. What can the builder do with his materials if both his hands and his tools are out of order or useless? It is exactly so with the infant. Destroy his digestion and he can do little or nothing with the best food materials in the world. The first thing now to do is to place him under thoroughly competent medical management, until his digesting tools are repaired and made perfect.

It is a poor account of the infantile stage of human existence which confines itself to the material aspects of the case. The doctor is more or less "cribb'd, cabin'd, and confined" within these narrow limits. But let no one forget that the baby has a mind; a mind to be amused and made happy; to be trained and disciplined. Not to be disciplined by severity. May all the angels of childhood forbid! But to be trained, developed, and disciplined by all that is best, sweetest, most reasonable, most patient, and most loving in gentle woman, or in devout and thoughtful man. If anything can bring out the best and noblest that is in man or woman, it is the presence and the possession of a child. Woe be to him who reverences not its helplessness, its innocence, and its trust! He is already far on his way to a fatal ending of his life's probation. Though we have mainly occupied our space with the conditions which affect the physical wellbeing of the infantile life, it is not that we have forgotten or that we attach little importance to other considerations. We hold that it is with childhood as with manhood: the body and the mind are inseparable, together they grow and develop; together they will fight life's battles; together they will come to victory and honour, or together to defeat and disgrace. Strange and deep-seeing words were those said so long ago, and still so strong and true: "Take heed that ye offend not one of these little ones: If any man offend one of these little ones . . . . It were better for him that a millstone were hanged about his neck, and that he were cast into the depths of the sea."

